# Nutrition Education Practices of Health Teachers from Shanghai K-12 Schools: The Current Status, Barriers and Willingness to Teach

**DOI:** 10.3390/ijerph17010086

**Published:** 2019-12-20

**Authors:** Fan Li, Yaqun Yuan, Xinming Xu, Jingsi Chen, Jiaxuan Li, Gengsheng He, Bo Chen

**Affiliations:** Key Laboratory of Public Health Safety of Ministry of Education, Collaborative Innovation Center of Social Risks Governance in Health, School of Public Health, Fudan University, Shanghai 200433, China; 16211020023@fudan.edu.cn (F.L.); yqyuan16@fudan.edu.cn (Y.Y.); 16301020028@fudan.edu.cn (X.X.); 15211020022@fudan.edu.cn (J.C.); 16301020034@fudan.edu.cn (J.L.); gshe@shmu.edu.cn (G.H.)

**Keywords:** nutrition education, health teacher, current status, barriers, willingness, teaching hours

## Abstract

China is facing challenges in both undernutrition and overnutrition, resulting from unhealthy diets. Nutrition education early in life, especially in school settings, has been reported to be effective in addressing these challenges. However, little is known about how nutrition education is delivered in schools in China. This study aimed to investigate the current status of delivering nutrition education by health teachers in Shanghai and to determine the barriers and resources that influence the teachers’ practices and their willingness to teach nutrition. In 2016–2017, a cross-sectional questionnaire survey was conducted on 904 health teachers from 823 K-12 schools in Shanghai, China. There were 722 (79.9%) teachers that had the experience of teaching nutrition, but only 137 (19.0% of 722) spent ≥1 h teaching nutrition courses in each school year. Only 18.6% of the teachers had received a formal education in nutrition in college. About 88.5% of teachers expressed their willingness to teach nutrition in the future. The three major reasons for never teaching nutrition were categorized as: nutrition being taught by other teachers (39.5%), willing to teach but lack of knowledge (37.9%), and the subject not being required by school administrators (31.3%). Teachers who spent more time or were more willing to teach nutrition courses were those who were female, from private schools, had a better background in receiving nutrition education, and were more concerned about nutrition. Our data show that nutrition education is at a formative stage in Shanghai, China.

## 1. Introduction

Over the past 40 years, China has undergone rapid economic growth, resulting in dramatic changes in the eating patterns of Chinese people [1]. China is currently facing challenges in both undernutrition and overnutrition. On the one hand, undernutrition is still present in vulnerable populations, especially those from poor areas. The 2014 data of the Chinese National Survey on Students’ Constitution and Health (CNSSCH) showed that the prevalence of undernutrition was 10.0% among Han students [2]. Undernutrition prevalence went up to 19.2% among children under five in the poor areas of China, according to the 2016 data from the United Nations International Children’s Emergency Fund (UNICEF) and the National Health and Family Planning Commission of China (NHFPC) [3]. On the other hand, the prevalence of both adult and childhood obesity in China has sharply increased in recent years. The China Health and Nutrition Survey (CHNS) reported that the prevalence of childhood (6–17 years) obesity had risen from 2.8% in 1991 to 10.1% in 2011 [4]. The obesity epidemic is one of the major reasons for obesity-related chronic diseases, which are typically observed in adults but also affect children and adolescents at increasing rates. According to CHNS data, the prevalence of hypertension in Chinese children and adolescents increased from 6.9 to 10.7% during 1991 to 2011 [5].

Unhealthy diets have been well-demonstrated to be the leading cause of the rising prevalence of obesity and its-related chronic diseases [6]. The 2010–2013 CHNS reported that Chinese people were use to a low consumption of vegetables, fruits, milks and bean products, and a high consumption of salts and oils [7]. Chinese students have been reported to have a high prevalence of skipping breakfast, eating desserts, and drinking sugar-sweetened beverages more than once per day [8,9,10,11].

One effective way to avoid unhealthy diets is to promote and establish healthy eating behaviors early in life [12,13]. School may be a good setting to help develop healthy eating behaviors. The school setting can teach nutrition and reinforce knowledge by providing food services, since most students spend plenty of time at school and consume at least one meal and several snacks at school each day [14,15,16]. Also, nutrition taught by teachers may be effective since they are authority figures who share knowledge with students [17]. Moreover, schools are important places to deliver nutrition-education-related policies, acts, or interventions [18]. School-based interventions, including the introduction of nutrition education into the school curriculum, have been shown to reduce overweight and obesity and increase students’ intakes of fruits and vegetables [19].

China has recognized the importance of nutrition education in its schools. The China State Council officially published the National Program for Food and Nutrition Development (NPFND, 2014–2020) on 10 Feb 2014 and set nutrition education as an important goal. This goal, as the China NPFND 2014–2020 announced in detail, is to incorporate food and nutrition knowledge into the curriculum of primary and secondary schools and to reinforce the nutrition education of teachers and parents [20].

Unfortunately, few studies have investigated the delivery of nutrition education in schools in China. The 2014 nutrition and health report of children and adolescents in China showed that only 10% of students had heard about “the Chinese Dietary Guideline (CDG)” or “the China Food Pagoda (CFP)” [21]. According to a Chinese report in 2012, there were 52.7% of health teachers from Shanghai that indicated that they lacked the ability to teach nutrition [22]. The current status of nutrition education practices in health teachers from China, and the barriers and limitations of resources they are facing, are mainly unknown. Those barriers, including nutrition knowledge, self-efficacy, willingness to teach, and supporting resources from school administrators, must be overcome to deliver nutrition education in schools in China.

Here, we performed a cross-sectional study of 904 health teachers from 823 K-12 schools in Shanghai by conducting a questionnaire survey. The study aimed to investigate the current status of delivering nutrition education for health teachers from Shanghai K-12 schools, to determine the barriers and resources that influence the teachers’ practices and their willingness to teach, and to investigate teacher-informed possibilities for incorporating future education in nutrition. These data from Shanghai can provide useful information to help understand nutrition education practices in China and also to contribute to policy development for the reinforcement of future practices.

## 2. Materials and Methods

### 2.1. The Enrollment of Schools and Participants

**Figure 1:**
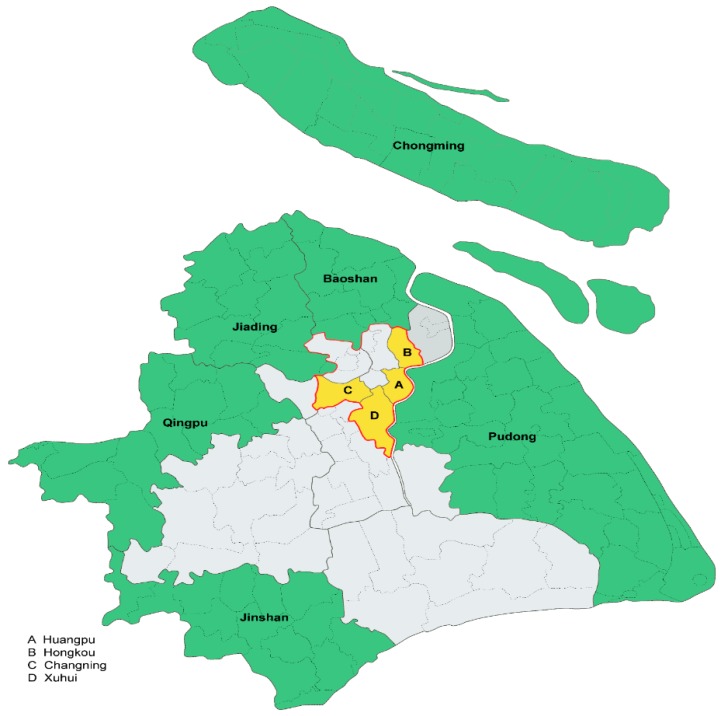
All health teachers from the K-12 schools of 10 districts of Shanghai city were invited to participate in this study. The 10 districts included four (Huangpu, Hongkou, Changning, and Xuhui) from the urban area and six (Chongming, Baoshan, Jiading, Qingpu, Jinshan, and Pudong) from the rural area.

To promote nutrition education in Shanghai schools (from kindergarten to grade 12, referred to as K-12 schools), the Shanghai Municipal Education Commission launched a program of training-to-trainers (TOT) in 2016. The program invited nutrition experts from colleges (Fudan University, Shanghai Jiao Tong University, and Shanghai Tongji University) or from Shanghai Center of Disease Control (SCDC) to give lecture courses to health teachers from K-12 schools. Generally, nutrition courses in K-12 schools from China are not compulsory and mainly taught by health teachers. The TOT program was organized by local Education Commission of 16 Shanghai districts, but only got the acceptance of local commission from 10 districts in September 2016 (Figure 1). This study was not part of the TOT program but was designed as an affiliated investigation by nutrition experts from Fudan University. The TOT program trained health teachers from other 4 districts in 2018, however these teachers were not investigated in this study. In total, there were 933 K-12 schools and 1037 health teachers from 10 investigated districts, averaging 1.11 health teachers per school. The K-12 schools from China are generally categorized into kindergarten school, primary school (K1–K5), middle school (K6–K9), high school (K10–K12), successive primary and middle school (K1–K9), successive middle and high school (K6–K12), successive primary, middle and high school (K1-K12), vocational and technical school. The successive schools are schools where students continue their study in the same school when they finish primary or middle school. In China, vocational and technical education is part of secondary education and offers vocational and technical training to students aged 16–18 years. The K-12 schools in China have two types: Public and private. The public schools are mainly opened to children of local citizens. The private schools also have two types: One is exclusive school with expensive tuition, another is opened to children of migrant workers. Generally, both public and private schools in China emphasize the entrance exams for higher-level schools. However, public schools and private schools for children of migrant workers are more examination-oriented, while exclusive schools are more quality-oriented. In 2016, the private schools in Shanghai were distributed as about 2/3 in exclusive schools and 1/3 in schools for children of migrant workers.

### 2.2. Questionnaire Survey

All health teachers were invited to attend six lectures of a three-day TOT program during October 2016 to January 2017. In one lecture on Chinese Dietary Guidelines, we invited health teachers to self-complete a 10-min WeChat-based questionnaire using their mobile phones. The questionnaire survey was conducted before the lecture was given when the teachers had arrived at the classroom. The WeChat software (Tencent, Shenzhen, China) is a messaging app that is capable of loading online questionnaire. For those without mobile phones or whose phones were unable to connect to WeChat, a paper questionnaire with the same content as the WeChat-based questionnaire was delivered to them to collect the information. In total, 890 participants answered the WeChat-based questionnaire and 44 answered the paper one. A total number of 934 health teachers (response rate: 90.1%) from 823 schools (response rate: 88.2%) agreed to participate in this cross-sectional survey. Written informed consent was obtained from all health teachers. The study was approved by the local authorities and the Ethics Committee of the School of Public Health at Fudan University (IRB#2016-TYSQ-03-4).

The questionnaire comprised of 15 closed-ended items including demographic information (gender, age, education level, school location, school type, school level, concerning about nutrition, and nutrition education background), questions about how health teachers delivered nutrition education in their school, and questions about the willingness, strategies, and detailed practices that may be helpful to promote future education in nutrition (Supplementary Materials). These question items were selected based on the literature review of both PubMed [23,24,25,26] and CNKI (China National Knowledge Infrastructure, the major network platform providing academic resources from China) [22,27,28]. The participants of the survey were not incentivized. The questionnaire has not been validated.

We designed four questions to collect information on current status of how health teachers deliver nutrition education. These questions included: (1) Do you have experience of teaching nutrition in the employed school?; (2) How many hours in each school year did you spend on teaching nutrition courses for each class of your students (<1, 1–2, 3–5, ≥6, or not sure)?; (3) If question (1) was answered with no, why had you never taught nutrition in the employed school (not being required by school administrators, nutrition was taught by other teachers, willing to teach but lack of nutrition knowledge, willing to teach but lack of school support, or other reasons)?; (4) Are you willing to teach nutrition in the future (willing, not willing, or not sure)?

With regard to the questions about willingness, strategies, and detailed practices that may be helpful to promote nutrition education, we first asked the willingness of health teachers to teach in the future (willing, not willing, or not sure). We then asked about the key strategies and possible practices that may be delivered by either the school administrators or the teachers themselves.

After the exclusion of 2 participants lacking of age information, 5 without nutrition education background, 12 not answering the questions of current performance of delivering nutrition education, 5 not answering the question of willingness to teach in the future, 3 not answering the question of key strategies, and 3 not answering the question of detail practices, 904 participants (87.2% of 1037) were ultimately included. Both included and excluded participants had no difference in demographic information (*p* < 0.05).

### 2.3. Statistical Analysis

The statistical analysis was performed using IBM SPSS Statistics for Windows Version 22.0 (IBM Corp., Armonk, NY, USA). Both a univariate logistic regression analysis and a multivariate logistic regression analysis were used to determine the potential demographic variables associated with (1) the current status of health teachers never teaching nutrition in the employed school, (2) the current status of health teachers teaching nutrition courses ≥1 h in their class in each school year, and (3) the willingness to teach nutrition in the future. School location in the questionnaire was listed as the names of 10 districts, but simplified as rural and urban areas for analysis (Figure 1). All demographic variables were forced into the multivariate regression models because they were suspected to influence the practices of delivering nutrition education based on either the literature review [23,26] or the results of the univariate analyses. Statistical significance was set at two-sided *p* < 0.05.

## 3. Results

### 3.1. Demographic Characteristics

Table 1 presents the demographic characteristics of 904 health teachers. The majority of them were female, teaching in public school, from a rural area, had a Bachelor’s degree or above, from kindergartens or primary schools, and highly concerned about nutrition. Few of them received formal education in nutrition in college.

### 3.2. Current Status of Delivering Nutrition Education by the Health Teachers

Table 2 presents the current status of how health teachers deliver nutrition education. A total of 722 (79.9%) teachers had the experience of teaching nutrition in their employed school, but most of them (73.7%) spent less than one hour per school year to teach nutrition courses. Among the 182 health teachers who never taught nutrition in their class, the top three reasons were as follows: Nutrition being taught by other teachers (39.5%), willing to teach but lack of knowledge (37.9%), and teaching nutrition not being required by school administrators (31.3%). About 88.5% of health teachers expressed their willingness to teach by running a nutrition course or in other ways.

### 3.3. Factors Associated with the Current Status of Delivering Nutrition Education

We used both univariate and multivariate logistic regression analyses to identify potential factors associated with teaching behavior (Table 3 and Table 4). Generally, a multivariate analysis attenuates the significance of the univariate analysis. By multivariate logistic analyses, health teachers who were male, with a higher school level, less concerned about nutrition, and had a poorer background of nutrition education, were less likely to teach nutrition (Table 3). By multivariate logistic analyses, the health teachers who spent more hours teaching nutrition courses were those from private schools rather than those from public schools, and those from primary schools or from successive middle and high schools rather than those from kindergartens (Table 4).

### 3.4. Factors That May Promote Nutrition Education in the Future: Willingness, Strategies, and Detailed Practices

There were 104 (11.5%) health teachers who were not willing to teach nutrition in the future or not sure whether they would be willing to (Table 2). We analyzed the factors associated with such willingness using both univariate and multivariate logistic regression models (Table 5). Teachers who were not willing (or not sure if they were willing) to teach were those from urban areas, normally or occasionally or rarely concerned about nutrition, and those never taught nutrition in the class. Teachers from private schools had a lower prevalence of not being willing (or not being sure if they were willing) to teach than those from public schools.

We also investigated the possible strategies in participants’ opinion that may promote nutrition education in K-12 schools in the future (Table 6). The top three key strategies were as follows: Being trained by nutrition experts (41.3%), providing resources for helping teach nutrition (26.5%), and setting nutrition lessons as a required course (16.5%).

We finally investigated the participants’ informed practices that could be possibly performed by either school administrators or health teachers themselves to deliver nutrition education (Table 7). The top three practices were as follows: Posting nutrition knowledge on bulletin boards in the classrooms and canteens (81.4%), integrating nutrition knowledge into health courses (72.7%), and posting nutrition knowledge on WeChat or Weibo (64.8%).

## 4. Discussion

This cross-sectional study investigated the current status of delivering nutrition education in Shanghai K-12 schools, determined the potential barriers to the process of delivering nutrition education, and explored the possible practices of future performance.

### 4.1. Current Status and Barriers of Delivering Nutrition Education in Shanghai

The China NPFND 2014–2020 recognized the importance of nutrition education for a healthy life [20]. School is a good setting to implement nutrition education due to its high accessibility to children and adolescents [14,15]. In this study, more than 88.0% of the participants responded that they were willing to teach nutrition, indicating that the majority of teachers were aware of the necessity of delivering nutrition education at an early age. In spite of this awareness, our data revealed that nutrition education in Shanghai was at formative stage and had a long way to go before delivering effective education.

Based on our study, although 79.9% of the teachers had taught some nutrition, only 26.3% of them spent ≥1 h per school year to teach nutrition courses. This figure was much lower than the national average of 13 h per school year (h/year) reported in the 2000 National Center for Education Statistics (NCES) of K-5 survey in America [29], and also much lower than the median value of 3.4 h/year of nutrition and dietary behavior instruction for elementary schools in America in 2006 [30]. It has been reported that a minimum of 15 h of nutrition instruction is required to initiate a change in knowledge and 50 h to retain lasting alteration in attitudes and behaviors [31]. Therefore, the teaching hours according to our study are far from being enough to deliver effective nutrition education.

The qualification of the teachers in Shanghai was questionable for delivering nutrition education. Less than 20.0% of the teachers in our study received formal education in nutrition in college, more teachers (44.7%) received occasional training courses, and 21.8% of the teachers never received training for nutrition education. It should be noted that educational background of occasional courses or lectures may not prepare teachers with the ability to deliver nutrition education to others. Even the extent of education background at the college level may have a wide range and may not fully prepare the teachers. The absence of training was not unique in our study. A survey in South Africa revealed that only 15.0% of the teachers had received nutrition training [32]. Insufficient formal training probably leads to the lack of nutrition knowledge, which in our data served as one of the barriers of not teaching nutrition. In another study from Shanghai, primary health teachers (*N* = 165) also reported high interest (86.1% of them were willing to offer nutrition instruction for the prevention of childhood obesity), but low qualification (52.7% indicated a lack of ability to teach nutrition) [22]. It was reported that providing training to trainers (e.g., teachers) had the potential to help students adhere to healthy diets [33,34] and prevent childhood overweight [35]. Yet, the challenges to deliver successful TOT programs may lie in the fact that the training opportunities are scarce and training providers are not available or qualified [36,37], as well as the teachers not having enough time or not being interested.

Schools are important places to deliver policies, acts, or interventions [18]. Studies have reported that the lack of understanding about the importance of nutrition education in school administrators was a significant barrier to deliver nutrition education [26,38], which is consistent with our findings. In our study, more than 31.3% of the teachers who never taught nutrition courses claimed that teaching nutrition was not required by school administrators. It is also necessary to recognize that lack of a requirement to teach may not reflect a lack of understanding the importance of nutrition education. For example, the entrance exams in China generally include traditional subjects of language, mathematics, etc., but not nutrition or food science; the school administrators may therefore emphasize the requirements of the traditional curriculum, but not nutrition or other subjects [39]. Health teachers from private schools in our data were more likely to teach longer or more willing to teach than those from public schools. This may be partially explained by the fact that the examination-oriented schools (public schools belong to this type) usually offer less courses on nutrition education than the exclusive private schools (belong to quality-oriented schools). Overall, both school administrators and teachers need to increase the perception of nutrition education by both recognizing the importance and setting up the requirements for detailed practices.

One major barrier for health teachers in providing nutrition instruction in our study was that other teachers were providing it. It should be noted that nutrition education in China is not delivered by specialized nutrition teachers, but by other teachers who teach health, biology, or even physical education [22,40,41]. Nevertheless, health teachers are the major figures to deliver nutrition education in China [22]. This was also the truth in our data, showing that 79.9% of health teachers had experience in teaching nutrition, but only 7.9% (72 of 904) of them did not teach it for the reason of other teachers doing it. Currently, nutrition is not the compulsory subject in China, and the delivery of nutrition education may depend on the interest of teachers in taking nutrition as part of their primary courses [22]. A good practice may be to increase the interest of teachers in nutrition instruction. Health teachers may be more eligible figures than other teachers since nutrition is closer to health than other subjects.

School level characteristic may be also an important factor to be addressed in association with nutrition education. Our study found health teachers from higher school levels were less likely to teach nutrition than those from kindergartens, although teachers from primary schools spent more hours teaching nutrition than the kindergarten teachers. Both students and teachers from examination-oriented schools in China, especially those from higher schools, face the pressure of entrance exams and therefore may possibly ignore the delivery of nutrition education [39]. One thing that needs to be addressed is which school level (kindergartens, primary schools, middle and high schools) may serve as the best setting for delivering nutrition education—this needs to be investigated for the implications of future practices and research.

Our study also identified health teachers who were male and less concerned about nutrition had poorer performance in delivering nutrition education. Given that most teachers were female (83.4%) and from kindergarten (41.4%), the sex difference on nutrition education in our data was biased by school level and therefore lacks value for implication. Teachers’ concern about nutrition represents one of the aspects of nutrition attitude, which has been reported to affect the delivery of nutrition education based on the general system theory [24]. This is important given that teachers’ attitude on nutrition may influence the attitude of students.

### 4.2. Implications for the Practice of Delivering Nutrition Education

Our study showed that more than 40% of the teachers considered being trained by nutrition experts as one of the key strategies to promote future performance. This finding was consistent with other studies where teachers admitted inadequacy at teaching nutrition and expressed their need for training despite many years of teaching experience [42,43]. Both our study and literature highlighted the importance of training teachers.

The government and school administrators should recognize the requirement of teaching nutrition and provide sufficient resources for helping the training of teachers. Taking South Korea as an example, the Korean Nutrition Teacher System was implemented in 2006 and started employing nutrition teachers since 2007 according to the Elementary and Secondary Education Act and School Meals Act [26]. Since then, 11,313 out of 11,575 schools had hired either nutrition teachers or school dietitians (49.6% of the total hires were nutrition teachers) according to the data from Korean Ministry of Education in 2013 [26]. The Korean nutrition teachers need to be qualified with both a teacher license as well as a dietitian license for providing meal services together with nutrition education [44]. Many universities in Korea opened special graduate programs to educate and train nutrition teachers [44]. Although the standards of nutrition education in Korean schools (such as minimum hours of classroom education or curriculum, etc.) have not been established, the nutrition teachers have tried various creative approaches (such as special class hours, discretional or club activities, experiential learning, etc.) [44]. The Korean Nutrition Teacher System is worth learning since China does not hire specialized nutrition teachers to deliver nutrition education in K-12 schools [22,40].

The topics of nutrition courses may include the knowledge about the relationship between diets and health, the Chinese dietary guidelines, the nutrients and their food sources, the methods of choosing and preparing healthy food, etc. [45]. These courses need plenty of time for the students in order to change knowledge, and more time to change attitudes and behaviors. Our data related to insufficient teaching hours on nutrition education in Shanghai health teachers highlighted the urgency for necessary practices. Meanwhile, it is also important to avoid a too-heavy workload for teachers, which has been regarded as one of the barriers to performing nutrition education [26].

It has been reported that teaching a nutrition course by either integrating it into other subjects or setting it as a separate subject enables nutrition information to reach learners [46]. According to the survey by U.S Department of Education, one-third of the teachers taught nutrition as a separate subject, while the same proportion integrated it into other subjects [29]. In our study, more teachers were willing to integrate nutrition education into health courses rather than offer an independent course. Compared to being integrated into other courses, nutrition education being offered as an independent course means the teachers probably spend more hours teaching it. Considering the finding of our study that the teaching hours in Shanghai K-12 schools were insufficient, setting a nutrition course as the independent subject may be a useful strategy to promote nutrition education.

Nutrition knowledge could be presented in varied forms including lectures, active classroom discussions, cooperative or collaborative work, as well as student projects, media presentations, role playing, etc. Resources including booklets, magazines, brochures, leaflets, posters, TV or radio channels, etc., may enhance the effective delivery of nutrition education in schools [15]. These resources help to create a school environment that is friendly for the students to reach nutrition messaging.

Additionally, nutrition education should not be limited to schools, but also include family and social network [47,48]. As referenced by China NPFND 2014–2020, reinforcing nutrition education in parents is also an important goal to be achieved [20]. Parents or grandparents who live with the children are usually their role models. When nutrition knowledge from school conflicts with what the students acquire from their parents or grandparents, such conflict may hinder the acceptance of new knowledge and its ability to modify behavior. Additionally, parents can request that their schools teach more nutrition, especially in the schools in which teachers have a high level of unwillingness to teach. The family support of nutrition education can be impaired or reinforced by social networks. Parents or grandparents are more likely to receive nutrition knowledge from media coverage than formal education. And in this case, the media coverage should provide correct and useful knowledge for delivering nutrition education to the family members. The social network may also facilitate nutrition education in schools. Social media may improve schools’ awareness of how important nutrition is and how it can be taught. Such awareness may facilitate the allocation of resources and benefit nutrition education.

### 4.3. Implications for Research on Delivering Nutrition Education

The findings of our study highlighted serval major implications for research needs on delivering nutrition education in schools in China.

First, research needs to investigate the current status and barriers of delivering nutrition education in different areas of China. Our findings revealed the insufficient teaching hours of nutrition courses in Shanghai K-12 schools. Given that Shanghai is the megacity of China and usually has more resources, those smaller cities and rural areas of China are possibly poorer in delivering nutrition education. Also, both students and teachers from these areas may have poorer knowledge, attitudes, and practices (KAP) with regard to nutrition education than those from megacities [28]. The successful delivery of nutrition education needs to address its current status and barriers in order to advance.

Second, research needs to assess the effectiveness of current and future education in nutrition in students. The short teaching hours of nutrition courses in our study was questionable for delivering effective nutrition education. Although the China NPFND set the goal of delivering nutrition education in schools, it did not include a nutrition curriculum for teachers to follow nor set the requirement for minimum hours of classroom curriculum [20]. There is lack of literature from China investigating such minimum hours for successful education in nutrition. Some intervention studies in K-12 school students from China reported better performance on the nutrition KAP after a short-term (typically months) but intensive education program [49,50]. It is important to know the effectiveness of delivering nutrition education across several aspects: (1) How long and how intensive of the education can be effective in changing the KAP (especially practices) of the students; (2) what forms of education, either independently or combined, can be effective; (3) how long can the change of better KAP be maintained after the end of successful education; (4) what is the difference of schools’ (or students’) characteristics on such effectiveness. Giving school-level characteristics as an example, the most cost-effective level needs to be identified so that the delivery of nutrition education can be emphasized in certain levels of schools (kindergarten, primary or middle schools, etc.).

Third, research needs to assess and develop a successful TOT program. Both our study and the literature highlighted the education or training requirement for the teachers [20,26]. The Korean Nutrition Teacher System is a good example by hiring nutrition teachers who received education training from universities through special graduate programs [44]. China may learn from Korea by hiring specialized nutrition teachers. If this cannot be done, China needs to train its own teachers (mainly health teachers) with effective programs. Currently, there are some localized TOT programs reported in our study and in the literature [51,52]. However, few of them have been assessed and generalized to country level.

Fourth, research needs to assess and develop policies for building effective school environments. The health teachers from our study identified two reasons for not teaching nutrition, including; the education not being required by school administrators and a lack of school support. School administrators are critical for a nutrition-education-friendly school environment to flourish [53]. While the China NPFND set the goal of school involvement, it did not offer detailed policies for schools to follow [20]. Once developed and validated by research, policies can be generalized to country level to help school administrators and teachers recognize the importance of nutrition education and effectively allocate the resources.

Fifth, research need to assess and develop strategies involving both school and family environment to deliver nutrition education. The China NPFND also set the goal of parental involvement in nutrition education [6], yet it was not examined in this study. Studies have found the combination of interventions addressing both school and family to be more effective than single setting [50]. Nutrition education needs to reach students, teachers, and parents as targets. Research is needed for the effect of school–family interaction on promoting nutrition education.

### 4.4. Limitations

The cross-sectional design of this study limits the deduction of casual associations. Only health teachers were sampled, while nutrition education might be delivered by other teachers. The study was based on a questionnaire survey in Shanghai, and could not be generalized to China. The questionnaire was not validated, and this may bias the results since the teachers might report fictive information. The questionnaire did not distinguish the types of private schools. There was no discussion on the district level characteristics beyond urban and rural location that could impact both supportive infrastructure and teacher ability and attitudes about providing nutrition instruction. The questions selected in our questionnaire were lacking some important items such as years of total teaching experience, and the forms and effectiveness of how nutrition knowledge was taught in the classroom, etc. The covariates of the study were heterogeneous and might require more information to help the analyses. In addition, the study used a closed-ended questionnaire to investigate the existing barriers of delivering nutrition education, but did not identify new social or cultural conditions that limited the practice. A mixed design of future study includes both quantitative and qualitative survey may help identify the new barriers.

## 5. Conclusions

This study provided a snapshot of nutrition education by health teachers in Shanghai, China and revealed that it was at a formative stage. Our data show that there is much room for the improvement of delivering nutrition education in China. Inadequacy of teaching hours and lack of training to teachers were revealed as factors limiting the delivery of nutrition education in Shanghai. These findings may help the development of nutrition education policies and intervention programs.

## Figures and Tables

**Table 1:** Demographic characteristics of health teachers (*N* = 904).

Variables	Total, *N* (%)
Sex	
Female	754 (83.4)
Male	150 (16.6)
Age (years)	40.6 ± 8.7
Education	
Bachelor’s degree or above	560 (62.0)
Junior college degree or below	344 (38.0)
School location	
Rural	596 (65.9)
Urban	308 (34.1)
School type	
Public	793 (87.7)
Private	111 (12.3)
School level	
Kindergarten	374 (41.4)
Primary school (K1–K5)	304 (33.6)
Middle school (K6–K9)	117 (13.0)
High school (K10–K12)	35 (3.9)
Successive primary and middle school (K1–K9)	35 (3.9)
Successive middle and high school (K6–K12)	23 (2.5)
Successive primary, middle and high school (K1–K12)	4 (0.4)
Vocational and technical school	12 (1.3)
Concern about nutrition	
Highly	620 (68.6)
Normally	269 (29.8)
Occasionally or rarely	15 (1.6)
Nutrition education background	
Formal education in nutrition in college	168 (18.6)
Occasional training course	404 (44.7)
Self-learning	90 (9.9)
Never received	197 (21.8)
Not remember	45 (5.0)

**Table 2:** Current status of how health teachers deliver nutrition education and their willingness to teach in the future (*N* = 904).

Variables	Total, *N* (%)
Had the experience of teaching nutrition in the employed school	
Yes	722 (79.9)
No	182 (20.1)
Hours of teaching nutrition courses in each school year (*n* = 722)	
<1	532 (73.7)
1–2	98 (13.6)
3–5	25 (3.5)
≥6	14 (1.9)
Not sure	53 (7.3)
Reasons for not teaching nutrition (multiple choices, *n* = 182)	
Not being required by school administrators	57 (31.3)
Nutrition was taught by other teachers	72 (39.5)
Willing to teach but lack of knowledge	69 (37.9)
Willing to teach but lack of school support	17 (9.3)
Other reasons	20 (11.0)
Willingness to teach nutrition in the future	
Willing	800 (88.5)
Not willing	8 (0.9)
Not sure	96 (10.6)

**Table 3:** Associated factors of health teachers not teaching nutrition (*N* = 904).

Variables	Univariate		Multivariate	
	OR (95%CI)	*p*	aOR * (95%CI)	*p*
Sex				
Female	Ref	-	Ref	-
Male	2.55 (1.73–3.75)	<0.001	2.10 (1.36–3.26)	0.001
Age	1.00 (0.98–1.02)	0.878	0.97 (0.95–1.00)	0.020
Education				
Bachelor degree or above	Ref	-	Ref	-
Junior college degree or below	0.81 (0.57–1.13)	0.216	0.86 (0.58–1.27)	0.445
School location				
Rural	Ref	-	Ref	-
Urban	0.86 (0.60–1.21)	0.381	0.98 (0.67–1.42)	0.906
School type				
Public	Ref	-	Ref	-
Private	0.86 (0.51–1.43)	0.553	1.04 (0.59–1.84)	0.895
School level				
Kindergarten	Ref	-	Ref	-
Primary school	2.28 (1.53–3.41)	<0.001	1.95 (1.25–3.03)	0.003
Middle school	2.29 (1.36–3.85)	0.002	1.81 (1.01–3.23)	0.047
High school	3.19 (1.47–6.93)	0.003	2.77 (1.18–6.49)	0.019
Successive primary and middle school	3.19 (1.47–6.93)	0.003	2.40 (1.05–5.45)	0.037
Successive middle and high school	2.46 (0.92–6.54)	0.072	2.07 (0.73–5.90)	0.174
Successive primary, middle, and high school	<0.01 (<0.01–∞)	0.999	<0.01 (<0.01–∞)	1.000
Vocational and technical school	2.32 (0.61–8.87)	0.219	1.89 (0.47–7.60)	0.372
Concern about nutrition				
Highly	Ref	-	Ref	-
Normally	2.18 (1.55–3.06)	<0.001	1.87 (1.30–2.68)	0.001
Occasionally or rarely	3.55 (1.24–10.20)	0.019	3.04 (0.97–9.46)	0.055
Nutrition education background				
Formal education in nutrition in-college	Ref	-	Ref	-
Occasional training course	2.68 (1.50–4.79)	0.001	2.11 (1.14–3.89)	0.017
Self-learning	1.72 (0.78–3.80)	0.178	1.32 (0.58–3.01)	0.515
Never received	4.26 (2.31–7.85)	<0.001	3.60 (1.86–6.96)	<0.001
Not remember	3.71 (1.59–8.65)	0.002	2.91 (1.18–7.16)	0.020
Constant	-	-	0.09	<0.001

* aOR: Adjusted odds ratio; all variables were forced into the models. Ref: Reference group. ∞: Infinite value.

**Table 4:** Associated factors of health teachers teaching nutrition courses ≥1 h per school year (*N* = 669).

Variables	Univariate		Multivariate	
	OR (95%CI)	*p*	aOR * (95%CI)	*p*
Sex				
Female	Ref	-	Ref	-
Male	1.35 (0.79–2.30)	0.273	1.13 (0.62–2.05)	0.694
Age	0.99 (0.96–1.01)	0.189	0.98 (0.95–1.00)	0.067
Education				
Bachelor degree or above	Ref	-	Ref	-
Junior college degree or below	0.88 (0.60–1.30)	0.524	0.85 (0.55–1.31)	0.462
School location				
Rural	Ref	-	Ref	-
Urban	1.04 (0.70–1.54)	0.856	0.91 (0.59–1.39)	0.653
School type				
Public	Ref	-	Ref	-
Private	1.69 (1.02–2.81)	0.042	1.99 (1.13–3.52)	0.018
School level				
Kindergarten	Ref	-	Ref	-
Primary school	1.84 (1.19–2.85)	0.006	2.26 (1.40–3.64)	0.001
Middle school	1.21 (0.64–2.29)	0.557	1.54 (0.77–3.08)	0.220
High school	0.89 (0.25–3.13)	0.853	1.15 (0.31–4.18)	0.838
Successive primary and middle school	2.13 (0.79–5.76)	0.136	2.76 (0.98–7.73)	0.054
Successive middle and high school	4.66 (1.62–13.44)	0.004	5.42 (1.77–16.6)	0.003
Successive primary, middle, and high school	<0.01 (<0.01–∞)	0.999	<0.01 (<0.01–∞)	0.999
Vocational and technical school	3.20 (0.74–13.81)	0.120	4.38 (0.94–20.33)	0.060
Concern about nutrition				
Highly	Ref	-	Ref	-
Normally	0.78 (0.50–1.22)	0.270	0.76 (0.47–1.22)	0.251
Occasionally or rarely	2.78 (0.61–12.60)	0.186	2.83 (0.57–13.99)	0.202
Nutrition education background				
Formal education in nutrition in college	Ref	-	Ref	-
Occasional training course	0.80 (0.50–1.30)	0.368	0.70 (0.42–1.17)	0.176
Self-learning	1.15 (0.60–2.20)	0.675	1.00 (0.50–1.99)	0.991
Never received	0.56 (0.30–1.05)	0.070	0.56 (0.29–1.09)	0.089
Not remember	1.09 (0.43–2.78)	0.860	0.95 (0.34–2.63)	0.923
Constant	-	-	0.32	0.120

* aOR: Adjusted odds ratio; all variables were forced into the models. Ref: Reference group. ∞: Infinite value.

**Table 5:** Associated factors of health teachers not willing (or not sure if they were willing) to teach (*N* = 904).

Variables	Univariate		Multivariate	
	OR (95%CI)	*p*	aOR * (95%CI)	*p*
Sex				
Female	Ref	-	Ref	-
Male	1.14 (0.67–1.94)	0.625	0.97 (0.52–1.81)	0.914
Age	1.00 (0.98–1.03)	0.888	0.99 (0.96–1.02)	0.389
Education				
Bachelor degree or above	Ref	-	Ref	-
Junior college degree or below	1.02 (0.67–1.55)	0.927	1.31 (0.81–2.12)	0.276
Location				
Rural	Ref	-	Ref	-
Urban	1.86 (1.23–2.81)	0.003	2.12 (1.36–3.32)	0.001
School type				
Public	Ref	-	Ref	-
Private	0.41 (0.17–0.95)	0.037	0.36 (0.14–0.89)	0.027
School level				
Kindergarten	Ref	-	Ref	-
Primary school	1.63 (1.02–2.60)	0.040	1.30 (0.76–2.21)	0.338
Middle school	1.17 (0.60–2.30)	0.640	1.06 (0.50–2.22)	0.882
High school	1.57 (0.57–4.28)	0.384	1.01 (0.33–3.09)	0.983
Successive primary and middle school	0.28 (0.04–2.08)	0.211	0.19 (0.02–1.54)	0.118
Successive middle and high school	1.41 (0.40–4.97)	0.595	1.50 (0.39–5.76)	0.557
Successive primary, middle and high school	<0.01 (<0.01–∞)	0.999	<0.01 (<0.01–∞)	0.999
Vocational and technical school	0.85 (0.11–6.80)	0.881	0.60 (0.07–5.03)	0.638
Concern about nutrition				
Highly	Ref	-	Ref	-
Normally	3.14 (2.04–4.83)	<0.001	3.07 (1.94–4.87)	<0.001
Occasionally or rarely	14.96 (5.19–43.17)	<0.001	17.33 (5.61–53.57)	<0.001
Nutrition education background				
Formal education in nutrition in-college	Ref	-	Ref	-
Occasional training courses	0.96 (0.54–1.70)	0.884	0.78 (0.41–1.46)	0.430
Self-learning	0.87 (0.38–2.01)	0.747	0.76 (0.30–1.91)	0.563
Never received	1.19 (0.63–2.24)	0.585	0.85 (0.41–1.75)	0.652
Not remember	1.21 (0.45–3.23)	0.708	0.73 (0.25–2.15)	0.572
Ever teach nutrition in the class	2.50 (1.61–3.87)	<0.001	2.19 (1.34–3.56)	0.002
Constant	-	-	0.09	0.005

* aOR: Adjusted odds ratio, all variables were forced into the models. Ref: Reference group. ∞: Infinite value.

**Table 6:** Key strategy that health teachers thought may promote the future nutrition education (*N* = 904).

Strategies	Key Strategy, *N* (%)
Being trained by nutrition experts	373 (41.3)
Providing resources for helping to teach nutrition	240 (26.5)
Setting nutrition lessons as a required course	149 (16.5)
Providing financial support on teaching nutrition	101 (11.2)
Others	30 (3.3)
Do not care	11 (1.2)

**Table 7:** Possible practices of nutrition education that health teachers thought could be performed in the future by either school administrators or themselves (*N* = 904) (multiple choices).

Practices	*N* (%)
Posting nutrition knowledge on bulletin boards in the classrooms and canteens	736 (81.4)
Integrating nutrition knowledge into health courses	657 (72.7)
Posting nutrition knowledge on WeChat or Weibo	586 (64.8)
Inviting nutrition experts to give lectures	562 (62.2)
Delivering nutrition materials (booklets, magazines, brochures, leaflets) to the students	559 (61.8)
Broadcasting nutrition knowledge in school-hosted TV or radio channels	546 (60.4)
Integrating nutrition knowledge into biology courses	300 (33.2)
Offering independent nutrition courses	280 (31.0)
Others	24 (2.7)
Do not care or not sure	39 (4.3)

## Supplementary Materials

The following are available online at https://www.mdpi.com/1660-4601/17/1/86/s1, Nutrition education questionnaire for health teachers from Shanghai K-12 schools.
