# Supplementary material for: Nutrition Education Practices of Health Teachers from Shanghai K-12 Schools: The Current Status, Barriers and Willingness to Teach
Source: Int J Environ Res Public Health. 2019 Dec 20;17(1):86. doi: 10.3390/ijerph17010086 (PMC6982291; doi:10.3390/ijerph17010086)
Supplement: Supplementary file 1 [file ijerph-17-00086-s001.pdf]

Nutrition education questionnaire for health teachers from Shanghai K-12 schools
(Questionnaire is in Chinese, and this is the translated version)

I. Demographic Information

1. Sex: _________
   1 Male
   2 Female

2. Date of Birth: _____Year _____Month _____Day

3. Education level: _________
   1 Junior college degree or below
   2 Bachelor degree
   3 Master degree or above

4. School location: _________
   1 Huangpu
   2 Baoshan
   3 Hongkou
   4 Jiading
   5 Changning
   6 Qingpu
   7 Pudong
   8 Chongming
   9 Jinshan
   10 Xuhui

5. School type: _________
   1 Public
   2 Private

6. School level: _________
   1 Kindergarten
   2 Primary school
   3 Middle school
   4 High school
   5 Successive primary and middle school
   6 Successive middle and high school
   7 Successive primary, middle and high school
   8 Vocational and technical school

7. To what extent do you concern about nutrition in daily life?
   1 Highly
   2 Normally
   3 Occasionally
   4 Rarely

8. Nutrition education background
   1 Systematic courses from college
   2 Occasional training course
   3 Self-learning
   4 No background
   5 Not remember

II. Nutrition Education Information

1. Do you have the experience of teaching nutrition in the employed school?
   1 Yes
   2 No (skip to Question 3)

2. How many hours in each school year did you spend on teaching nutrition for each class of your students?
   1 < 1 hour
   2 1-2 hours
   3 3-5 hours
   4 ≥ 6 hours
   5 Not sure

3. Why you never taught nutrition in the employed school?
   1 Not being required by school administrators
   2 Nutrition was taught by other teachers
   3 Willing to teach but lack of nutrition knowledge
   4 Willing to teach but lack of school support
   5 Other reasons

4. Are you willing to teach nutrition in the future?
   1 Willing
   2 Not willing
   3 Not sure

5. Which of the following do you think is the key strategy to promote the future nutrition education?
    1 Setting nutrition lessons as a required course
    2 Providing resources for helping teach nutrition
    3 Providing financial support on teaching nutrition
    4 Being trained by nutrition experts
    5 Others
    6 Not care

6. In addition to the above key strategy, what else strategies do you think may promote the future nutrition education? (multiple choices)
    1 Setting nutrition lessons as a required course
    2 Providing resources for helping teach nutrition
    3 Providing financial support on teaching nutrition
    4 Being trained by nutrition experts
    5 Others
    6 Not care

7. Which of the following practices of nutrition education do you think may performed by either school administrators or yourselves in the future? (multiple choices)
    1 Offering independent nutrition courses
    2 Integrating nutrition knowledge into health courses
    3 Integrating nutrition knowledge into biological courses
    4 Posting nutrition knowledge on WeChat or Weibo
    5 Posting nutrition knowledge on bulletin boards in the classrooms and canteens
    6 Inviting nutrition experts to give lectures
    7 Delivering nutrition materials (booklets, magazines, brochures, leaflets) to the students
    8 Broadcasting nutrition knowledge in school-hosted TV or radio channels
    9 Others
    10 Do not care or not sure
